# Integrative Predictive Nomograms for Treatment Decision-Making in Resectable Synchronous Colorectal Liver Metastases

**DOI:** 10.7150/jca.107194

**Published:** 2025-01-27

**Authors:** Yujuan Jiang, Dedi Jiang, Jinghua Chen, Heting Feng, Zixing Zhu, Jun Jiang, Fan Wu, Jianwei Liang

**Affiliations:** 1 Department of Colorectal Surgery, National Cancer Center/National Clinical Research Center for Cancer/Cancer Hospital, Chinese Academy of Medical Sciences and Peking Union Medical College, Beijing, China.; 2 Department of Pancreatic and Gastric Surgery, National Cancer Center/National Clinical Research Center for Cancer/Cancer Hospital, Chinese Academy of Medical Sciences and Peking Union Medical College, Beijing, China.; 3 Department of Hepatobiliary Surgery, National Cancer Center/National Clinical Research Center for Cancer/Cancer Hospital, Chinese Academy of Medical Sciences and Peking Union Medical College, Beijing, China.; 4 Department of Diagnostic Radiology, National Cancer Center/National Clinical Research Center for Cancer/Cancer Hospital, Chinese Academy of Medical Sciences and Peking Union Medical College, Beijing, China.; 5 Department of Plastic and Cosmetic Surgery, Tongji Hospital, Tongji Medical College, Huazhong University of Science and Technology, Wuhan, China.

**Keywords:** CRLM, upfront surgery, neoadjuvant therapy, prognostic models

## Abstract

**Background:** Currently, there is no established standard for managing resectable synchronous colorectal liver metastases (CRLM): upfront surgery or neoadjuvant therapy. This study has integrated four available clinical factors - clinicopathological characteristics, gene mutation profiles, imaging findings, and hematological indicators - to create a potentially robust tool aiding clinicians in deciding between upfront surgery and neoadjuvant therapy.

**Methods:** This retrospective cohort study included individuals diagnosed with resectable synchronous CRLM between 2008 and 2018. The development of prediction nomograms entailed identifying independent prognostic indicators through univariate and multivariate Cox analyses. The accuracy of the predictions was evaluated through calibration curves and the C-index. Furthermore, the clinical effectiveness of the nomograms was assessed using DCA and ROC curves. To enhance accessibility, two web servers were developed to simplify the utilization of the nomograms for an improved user experience.

**Results:** A total of 386 patients with resectable synchronous CRLM were included. The patients were categorized randomly into a training cohort (n = 270, 70%) and a testing cohort (n = 116, 30%). The nomograms incorporated nine predictors: metastatic tumor count, cN stage, KRAS and BRAF mutation status, age, primary tumor location, neutrophil and platelet counts, and D-Dimer levels. The calibration plots for resectable synchronous CRLM survival predictions showed remarkable consistency. The C-index of OS and DFS prediction models were both above 0.7. And the area under the ROC curve of 1-, 3- and 5-year OS and DFS exceeded 0.7 as well. As demonstrated by the DCA plots, both nomograms exhibit satisfactory clinical effectiveness. A web-based application was developed to demonstrate the practical application of the prediction models.

**Conclusion:** The personalized web-based predictive models exhibited moderate predictive accuracy in resectable synchronous CRLM. These tools offer valuable assistance to physicians in deciding between upfront surgery and neoadjuvant therapy for resectable synchronous CRLM.

## Introduction

Colorectal cancer (CRC) ranks as the third most common cancer globally, with the second-highest mortality rate^1^. The liver is the most prevalent site for CRC metastases, presenting a challenging prognosis for patients, as evidenced by a 5-year survival rate of less than 10%^1^. Liver resection is the recommended strategy for patients with colorectal liver metastases (CRLM), endorsed clinically and by the National Comprehensive Cancer Network (NCCN) guidelines as the optimal approach for long-term survival and potential cure^2^. Moreover, preoperative therapy offers a spectrum of potential advantages, such as timely intervention for micrometastases, assessment of treatment efficacy, and the avoidance of local therapy for patients exhibiting early disease progression indicators^2^-^4^. However, the choice between neoadjuvant therapies and upfront surgery remains contentious.

The European Society for Medical Oncology (ESMO)^5^ recommends that oncological (prognostic) and technical (surgical) criteria be taken into account when evaluating upfront surgery or neoadjuvant treatment. However, neither ESMO nor NCCN provides definitive screening criteria^2^, ^5^, which results in a heavy reliance on the doctor's expertise. Consequently, there is a growing trend towards risk stratification and the identification of “high-risk” patients^6^. However, persistent issues remain in the field of predictive modeling. Some models only present the variables identified in screening without undergoing validation^7^-^9^ while others focus exclusively on clinicopathological features, overlooking important predictors such as the status of KRAS and BRAF mutations^10^.

In addressing the above clinical concerns, this study constructed and validated two evolving preoperative predictive models. It scrutinized nine variables across four domains encompassing clinicopathological features, gene mutation status, imaging evidence, and hematological parameters. By computing patients' survival risk scores, this approach can potentially aid physicians in determining the suitability of upfront surgery for resectable synchronous CRLM.

## Materials and Methods

### Patient selection

**Figure 1:**
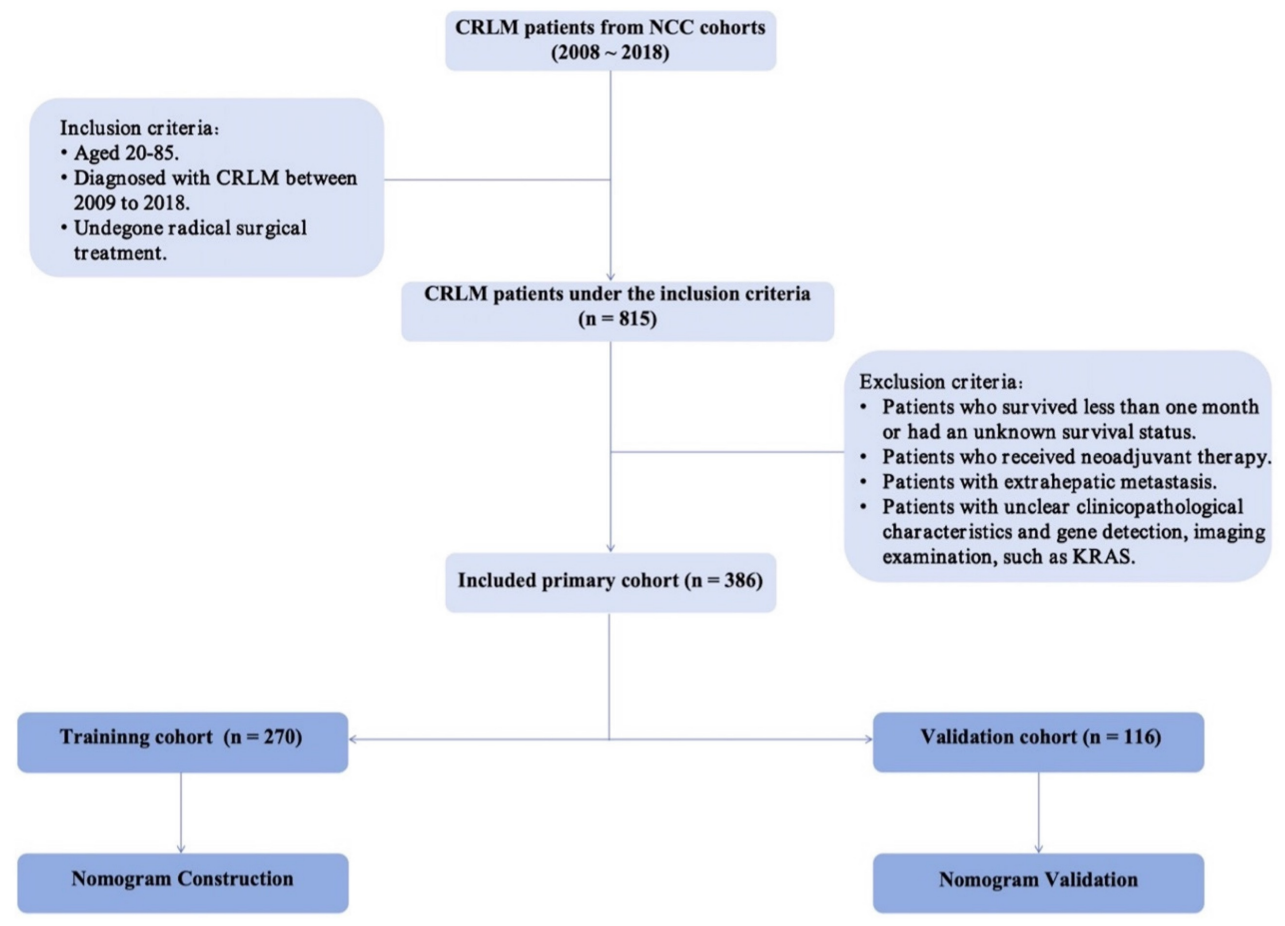
Screening procedure for enrolled CRLM patients.

A total of 815 patients diagnosed with CRLM between 2008 and 2018 at the National Cancer Center were included in this retrospective cohort study. Eligibility criteria comprised the following: (1) aged between 20 and 85 years, (2) diagnosis of resectable synchronous CRLM between 2008 and 2018, and (3) undergone radical surgical intervention. Exclusion criteria entailed (1) patients with unknown survival status, (2) individuals who underwent neoadjuvant therapy, (3) patients with extrahepatic metastasis, and (4) patients lacking clear clinicopathological characteristics, imaging examinations, or gene mutation information. A total of 386 patients were recruited for the study. ***Figure 1*** shows the patient screening procedure. The research was retrospectively registered. Patient information was obtained by telephone follow-up (once a year) or review records. The National Cancer Center's Institute Research Medical Ethics Committee approved this study (NCC2021C-125). Following review by the National Cancer Center's Institute Research Medical Ethics Committee, our study was granted an exemption from obtaining signed informed consent.

### Prognostic variables

The study gathered data on various parameters including the size of the largest metastatic tumor, number of metastatic tumors, clinical T(cT) and N(cN) stages, KRAS, NRAS, and BRAF statuses, gender, age, body mass index (BMI), American Society of Anesthesiologists (ASA) physical status classification, tumor grade, histological type, primary tumor site, pretreatment carcinoembryonic antigen (CEA) levels, neutrophil, lymphocyte, monocyte, and platelet counts, neutrophil-to-lymphocyte ratio (NLR), platelet-to-lymphocyte ratio (PLR), C-reactive protein (CRP), albumin (Alb), Glasgow Prognostic Score (GPS), red blood cell distribution width (RDW-CV), and D-Dimer levels. The primary outcome of the study was overall survival (OS), defined as the period from the date of diagnosis until death from any cause or the last follow-up. Disease-free survival (DFS) was designated as the secondary endpoint. Patients were divided into two age groups: <70 years old and ≥70 years old. The patients were then categorized based on the following criteria: the largest size of metastatic tumors (<2.25cm and≥2.25cm), the number of metastatic tumors (<3 and≥3), CEA levels (<16.110ng/ml and≥16.110ng/ml), neutrophil count (<3.435*10^9^ and≥3.435*10^9^), lymphocyte count (<2.795*10^9^ and≥2.795*10^9^), monocyte count (<0.545*10^9^ and ≥0.545*10^9^), platelet count (<282.5*10^9^ and≥282.5*10^9^), NLR(<2.677 and≥2.677), PLR(<130.481 and≥130.481), CRP levels (<0.085mg/L and ≥0.085mg/L), albumin levels (<44.95g/dl and ≥44.95g/dl), RDW-CV (<15.35 and ≥15.35), and DD levels (<0.545mg/L and ≥0.545mg/L), based on the optimal cut-off value obtained from the receiver operating characteristic (ROC) curve (refer to ***Supplementary Figure 1***).

### Statistical analysis

Statistical analysis was conducted utilizing the R program, version 4.3.2, available for download at https://www.r-project.org/. The patients were randomly assigned: 70% were designated to the training cohort, and the remaining 30% were allocated to the validation cohort, utilizing the “caret” package in R. Categorical variables are shown as numbers and percentages. Variables with a significance level of P<0.1 were initially selected using univariate Cox regression analysis, followed by multivariate Cox regression analysis to identify independent factors (P<0.05) influencing OS and DFS. Subsequently, two prognostic nomograms were developed, utilizing distinct predictive variables to predict OS and DFS in patients with resectable synchronous CRLM. Dynamic nomogram models were developed using the “rms” and “Dynnom” packages. We employed the Harrell consistency index (C-index), calibration curve, Receiver operating characteristic (ROC) curves and decision curve analysis (DCA) to assess the model's accuracy and appraise its clinical utility. Based on the prediction model, the risk score for each patient was computed, and the optimal cut-off value from the ROC curve was utilized to stratify patients into high-risk and low-risk groups. Subsequently, Kaplan-Meier survival curves were employed to assess the disparities in OS and DFS between these groups. A significance level of p < 0.05 (two-tailed) was applied to all statistical tests.

## Results

### Patient characteristics

This study included a total of 386 patients with resectable synchronous CRLM, among whom 270 patients (70%) were randomly allocated to the training group, with the remaining 116 patients (30%) assigned to the test group. The median OS and DFS for the entire patient cohort were 40.50 months (IQR, 23.25-61.00) and 25 months (IQR, 9.25-55), respectively. Table 1 displays the detailed data. The majority of patients (57.3%) were under the age of 70. Adenocarcinoma (98.7%) was the predominant pathological type, mostly highly differentiated. Most patients (74.1%) tested negative for the KRAS gene, with similar findings on the NRAS and BRAF genes. Among the sample, 281 individuals (72.8%) had fewer than three metastases. The most common cT stage observed was T3, accounting for 213 cases (55.2%). The left colon was the primary site for 289 patients (74.9%).

### Independent influencing factors of OS and DFS were selected

The univariate Cox regression analysis revealed that the number of metastatic tumors, cN stage, KRAS and BRAF gene mutations, patient age, primary tumor site, CEA levels, neutrophil count, platelet count, and D-Dimer levels exhibited a significance level of *P* <0.1. Hence, these variables demonstrated a significant correlation with patients' OS. The results of the multivariate analysis indicated that the number of metastatic tumors (HR 2.46, *P* < 0.001), cN stage (*P* < 0.001), KRAS mutation (HR1.66, *P* < 0.05), BRAF mutation (HR 4.58, *P* < 0.001), age (HR 1.62, *P* < 0.05), primary tumor site (HR 1.61, *P* < 0.05), neutrophil count (HR 1.80, *P* < 0.05), platelet count (HR 2.53, *P* < 0.05), and D-Dimer levels (HR 1.62, *P* < 0.05) were the factors significantly associated with OS (***Table 2***). ***Supplementary Figure 2*** presents a forest plot illustrating the HRs and 95% CIs for OS determined through Cox proportional hazards regression analysis.

In predicting DFS, factors such as the number of metastatic tumors, cN stage, KRAS and BRAF mutations, patient age, primary tumor site, CEA levels, neutrophil count, monocyte count, platelet count, and D-Dimer levels were identified as significant predictors for DFS using univariate analysis. The subsequent multivariate analysis revealed that the number of metastatic tumors (HR 2.48, *P* < 0.001), cN stage (HR 3.32, *P* < 0.001), KRAS (HR 1.74, *P* < 0.05), BRAF (HR 2.81, *P* < 0.001), age (HR, *P* < 0.05), original site (HR 1.83, *P* < 0.05), neutrophil count (HR 1.61, *P* < 0.05), platelet count (HR 2.58, *P* < 0.05), D-Dimer (HR 1.62, *P* < 0.05) were identified as independent predictors. These findings are presented in ***Table 3.*** Moreover, a forest plot from the multivariate regression analysis for DFS was generated in ***Supplementary Figure 3***.

### Development of nomograms for OS and DFS

**Figure 2:**
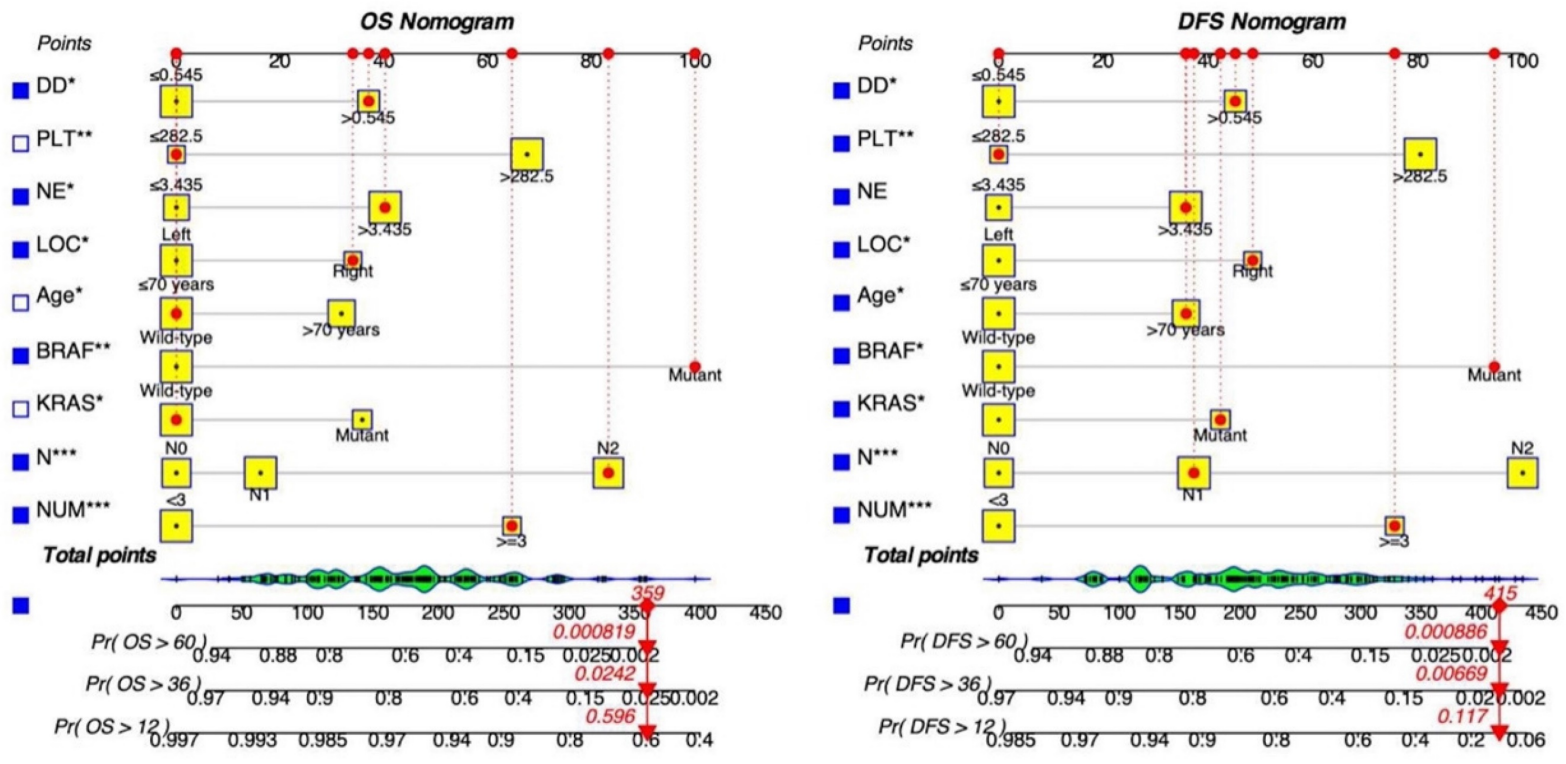
** Dynamic nomograms for predicting the prognosis of patients with resectable synchronous CRLM.** Distinct variables are associated with specific scores, and the cumulative scores correspond to the 1-year, 3-year, and 5-year survival rates.

Key screening indicators, including the number of metastatic tumors, cN stage, mutations in KRAS and BRAF genes, patient age, primary tumor site, neutrophil count, platelet count, and D-Dimer levels, were integrated into the dynamic prediction model to forecast OS and DFS in patients with resectable synchronous CRLM. ***Figure 2*** illustrates the process of determining a patient's score for each variable by intersecting a vertical line with the factor value score line. The cumulative total score corresponds to various survival rates. This visual model enables an intuitive assessment of the likelihood of 1-year, 3-year, and 5-year survival as well as disease-free survival.

**Figure 3:**
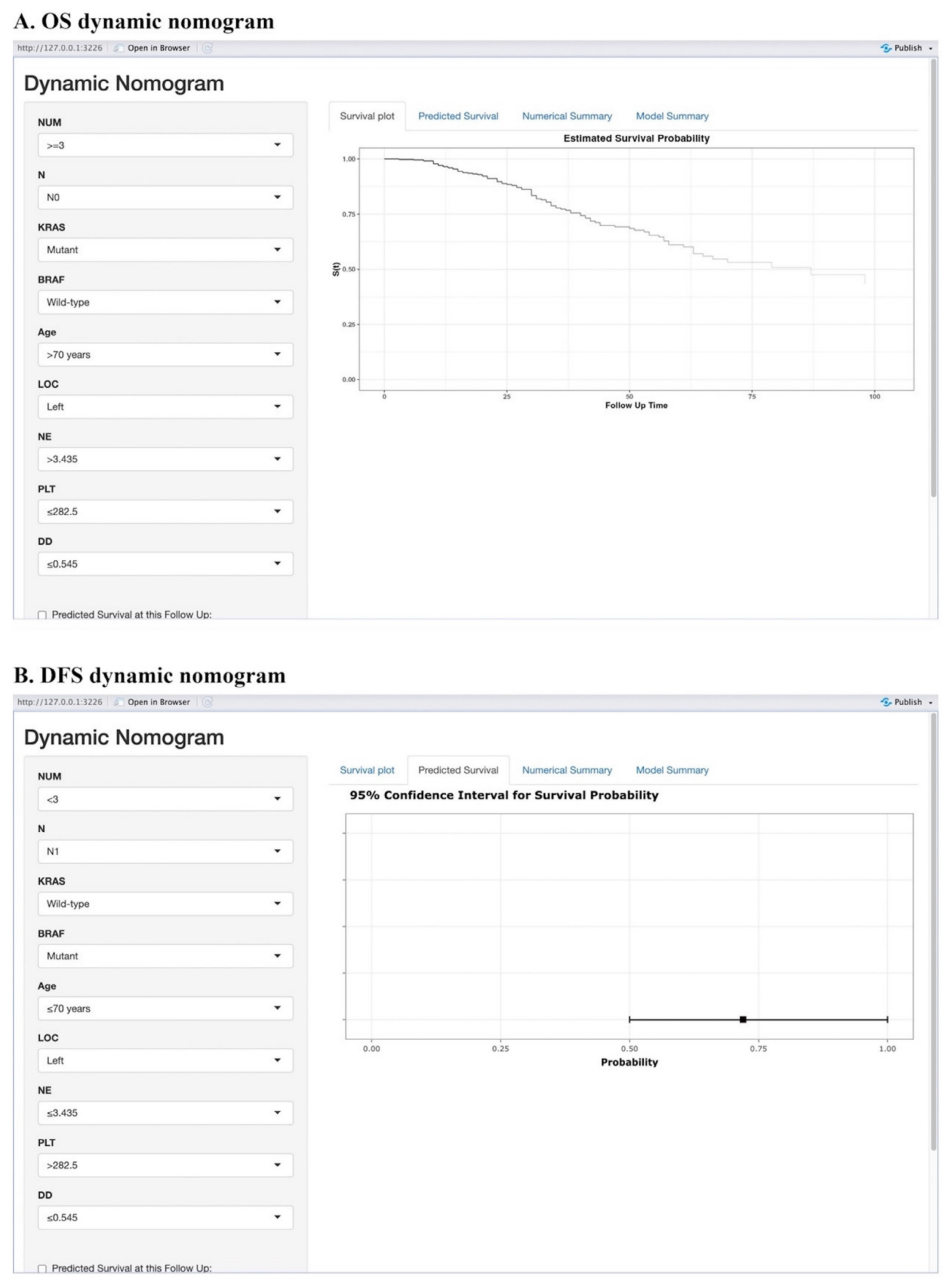
** Web-based prognostic nomogram for patients with resectable synchronous CRLM.** Available at: https://alxpcun.shinyapps.io/OS-nomogram/ and https://alxpcun.shinyapps.io/DFS-nomogram/.

Moreover, we have created an interactive online application featuring the generated nomograms (Refer to ***Figure 3***). The hyperlinks (https://alxpcun.shinyapps.io/OS-nomogram/and https://alxpcun.shinyapps.io/DFS-nomogram/)are accessible for navigation.

### Validation of the nomograms

**Figure 4:**
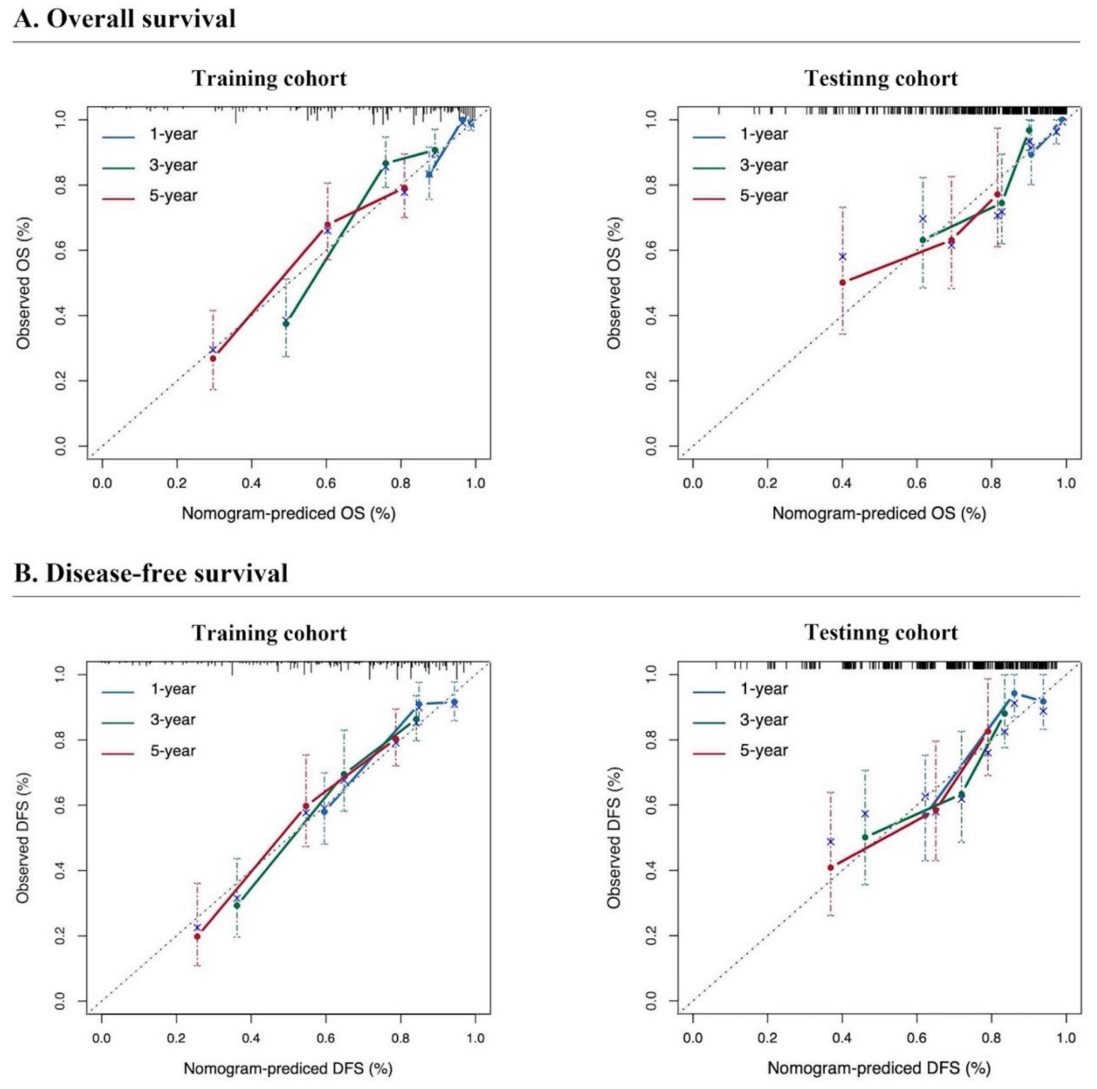
** Calibration curves for predicting the survival of resectable synchronous CRLM patients.** The calibration curve of the prediction model closely approximates the reference line, signifying a strong alignment between the predicted and actual probabilities.

**Figure 5:**
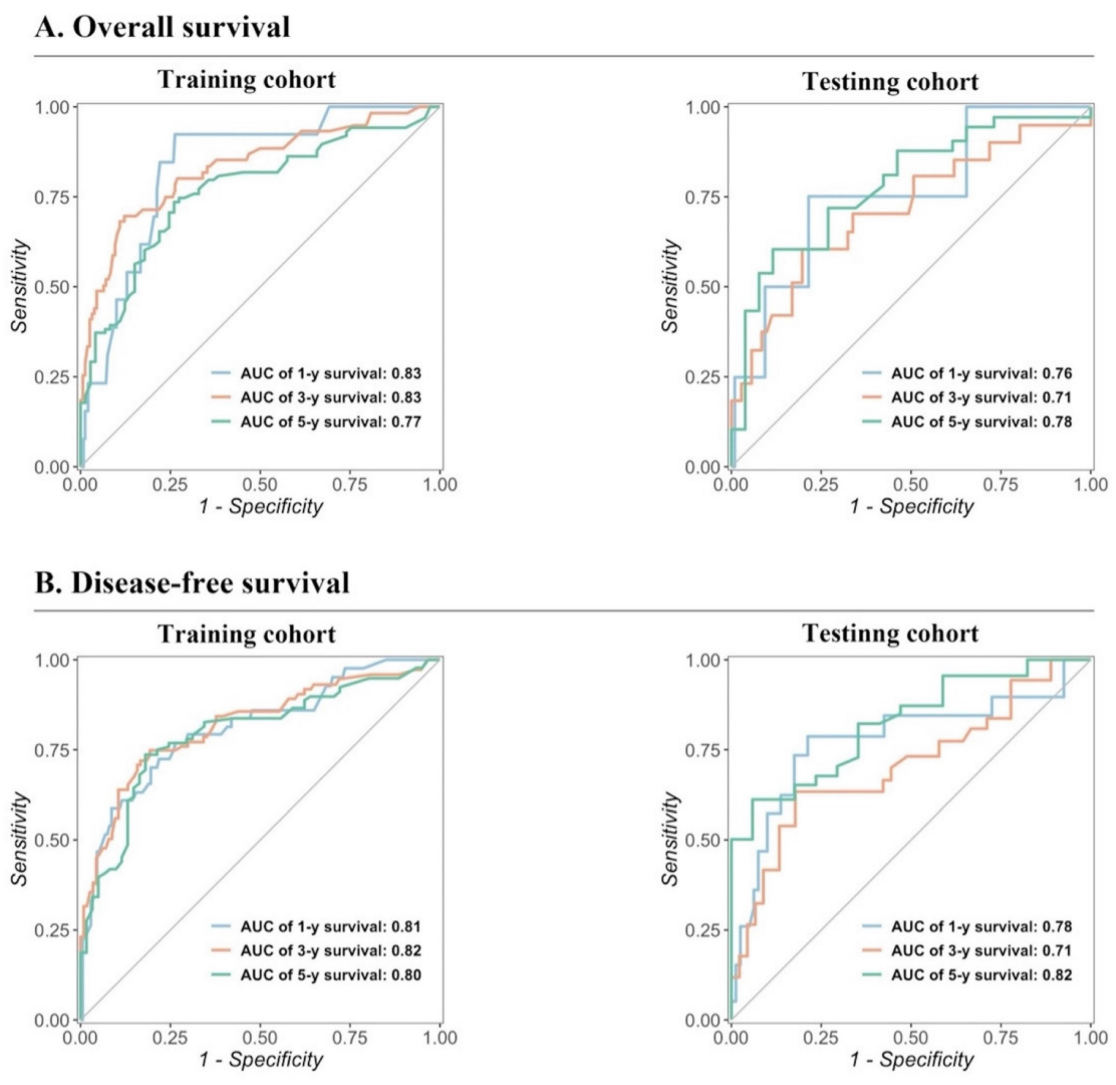
Validation of the prognostic nomograms using ROC curves.

The predictive performance of the models in both the training and testing cohorts was evaluated through calibration plots, C-index, AUC, and DCA. Initially, calibration curves were generated to compare the projected 1-, 3-, and 5-year OS and DFS probabilities from the nomogram models with the actual outcomes of resectable synchronous CRLM patients in the training and testing sets. The results demonstrated strong concordance between the predicted probabilities and the observed outcomes, validating a high level of accuracy in the predictions, as depicted in ***Figure 4***. The nomogram exhibited substantial precision in survival prediction, with C-index values of 0.773 ± 0.05 and 0.764 ± 0.053 for OS and DFS, respectively, in the training cohort. In the testing group, the C-index values were 0.747 ± 0.085 and 0.741 ± 0.082 for OS and DFS, respectively. ROC curves were constructed to evaluate the sensitivity and specificity of the nomogram prediction models. The training cohort displayed AUC values of 0.83, 0.83, and 0.77 for OS at 1, 3, and 5 years, respectively, as well as AUC values of 0.81, 0.82, and 0.80 for DFS at the corresponding time points. Remarkably, the testing cohort displayed AUC values of 0.76, 0.71, and 0.78 for OS and 0.78, 0.71, and 0.82 for DFS at 1, 3, and 5 years respectively, as illustrated in*** Figure 5***. In summary, our data validated the high sensitivity and specificity of our nomogram models.

**Figure 6:**
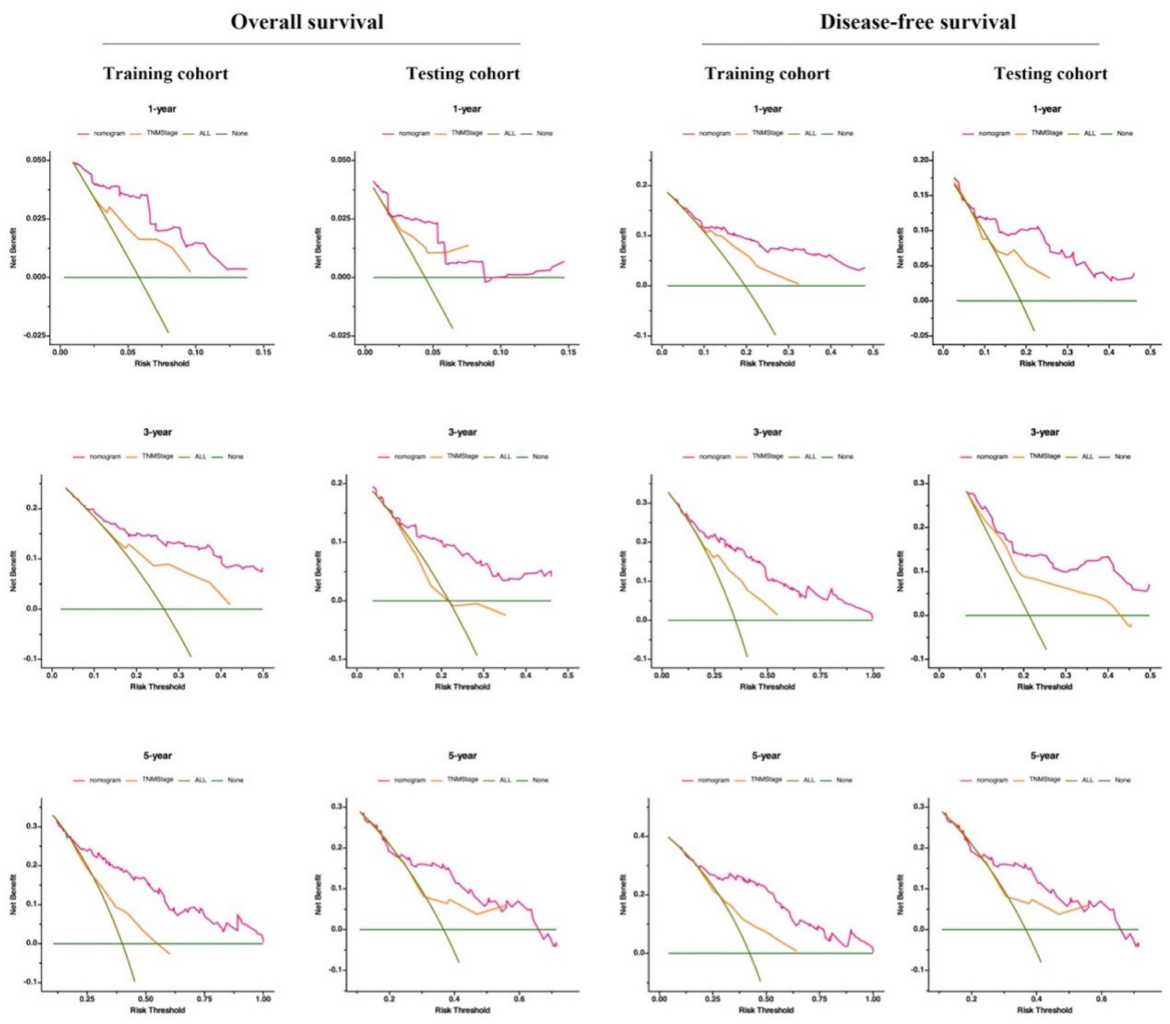
** Validation of the prognostic nomograms using DCA curves.** ALL, all patients died or relapsed; none, no patients died or relapsed. The net benefit rate of the nomogram curve surpasses those of the TNM stage and extreme curve, highlighting the substantial practical utility of prediction models.

Additionally, DCA serves as a widely adopted method for evaluating the clinical usefulness of nomograms. The nomograms surpass the conventional TNM staging system, demonstrating superior predictive capabilities for mortality risk, as depicted in ***Figure 6***. These results emphasize the considerable practical importance of nomograms in predicting OS and DFS for patients diagnosed with resectable synchronous CRLM.

### Risk status of patients stratified by the prediction models

**Figure 7:**
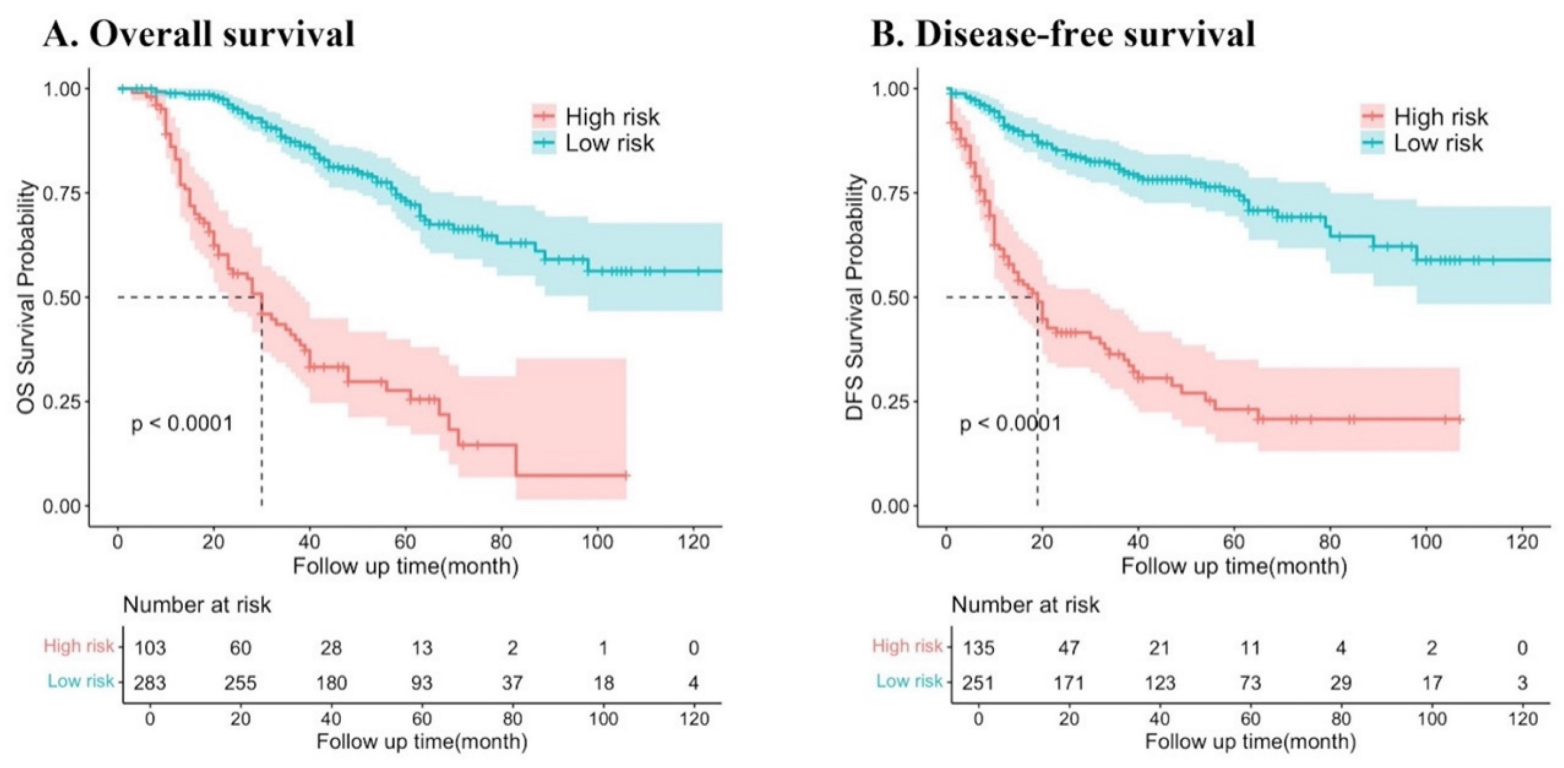
Kaplan‒Meier survival curves of resectable synchronous CRLM patients at different risk factors for death.

The predictor variable scores were calculated using the nomogram and then totaled to determine the cumulative scores for each patient. Patients with resectable synchronous CRLM were stratified into low- and high-risk categories based on their nomogram scores. The threshold score was set at 295.4 points for DFS and 283.5 points for OS on the nomogram. Patients exceeding the threshold were classified as high risk. Survival analysis revealed a significantly lower probability of DFS and OS in the high-risk group compared to the low-risk group (both P < 0.001). These results indicate that the nomograms utilized in this study can effectively stratify the risk for resectable synchronous CRLM patients (***Figure 7***).

## Discussion

NCCN and ESMO guidelines endorse surgical intervention or perioperative (neoadjuvant plus postoperative) systemic therapy for patients with resectable synchronous colorectal liver metastases (CRLM)^2^, ^5^. However, the optimal sequence for administering systemic therapy and resection remains uncertain. The decision between neoadjuvant therapy and upfront surgery for resectable synchronous CRLM varies greatly among medical centers and is largely subjective. Present predictive models for resectable synchronous CRLM predominantly focus on postoperative survival prognostication rather than assisting in the choice between upfront surgery or neoadjuvant therapy. They also disregard vital hematological and genetic factors, such as neutrophil count, platelet count, and KRAS status, which are known to significantly influence patient survival^11^, ^12^. Thus, further research is imperative to investigate the factors influencing the prolonged survival of resectable synchronous CRLM patients and to create predictive models for optimizing treatment selection.

In order to enhance the comprehensiveness and accuracy of the model, this study introduced four indices - clinicopathological features, genetic status, imaging, and hematological factors - for the first time. Subsequently, univariate and multivariate Cox regression analyses were conducted using comprehensive clinical data to identify independent risk variables for the prognosis of OS and DFS in patients with resectable synchronous CRLM. Nine prognostic factors were then identified: metastatic tumor count, cN stage, KRAS and BRAF status, patient age, primary tumor location, neutrophil and platelet counts, and D-Dimer level. Furthermore, an interactive online tool was developed to expedite clinical decision-making based on the provided nomograms. The calibration of the nomograms demonstrated strong performance, with the OS and DFS models exhibiting a C-index and AUC greater than 0.7, indicating their robust discriminative ability. Additionally, DCA illustrated that our novel nomogram models offered superior clinical utility compared to the traditional TNM staging system across various threshold probabilities. These results suggest that our nomograms could aid clinicians in deciding between upfront surgery and neoadjuvant therapy.

In recent years, various studies have highlighted the significance of the primary tumor site, lymph node metastasis status, age, and the number of metastatic tumors as key prognostic factors for patients with resectable synchronous CRLM, leading to their incorporation into clinical prediction models^13^, ^14^. Our research aligns with these findings. Although there are differences in how these variables are grouped, they consistently emerge as independent risk factors affecting the outcomes of resectable synchronous CRLM patients. Busiman *et al.*^15^ integrated several factors including age, gender, location and nodal status of CRC, disease-free interval, number and size of CRLM, preoperative CEA levels, resection margin, presence of extrahepatic disease, KRAS and BRAF mutation status, histopathological growth pattern, perioperative systemic chemotherapy, and perioperative hepatic arterial infusion pump (HAIP) chemotherapy into their clinical prediction model using Cox regression analysis, yielding satisfactory predictive performance. It is noteworthy that their model focused on predicting the 10-year survival post-surgery for patients with CRLM, rather than aiding in preoperative surgical decision-making, and did not consider the predictive value of hematological factors. These limitations are also prevalent in other related studies^16^-^18^.

Genetic factors, such as the status of BRAF and KRAS, significantly impact the treatment recommendations for patients with resectable synchronous CRLM^11^, ^19^. Previous models were limited by data availability, hindering the incorporation of all relevant variables. Our models emphasize the crucial roles of KRAS and BRAF in predictive modeling. Mutations in KRAS and BRAF promote cancer progression by enhancing angiogenesis, influencing cell motility and adhesion, and triggering aggressive biological traits in cancer cells^20^, ^21^. As a result, patients demonstrate significantly decreased OS and early tumor recurrence. Our findings are consistent with the study by Huang *et al.*^22^, confirming that KRAS and BRAF mutations act independently as prognostic risk factors in resectable synchronous CRLM patients. While the mutation status significantly influenced the outcome in the prediction model, the mutation rates of KRAS and BRAF were notably low, diminishing their clinical predictive value^23^. Additionally, increasing research supports the use of hematological markers such as neutrophil count, platelet count, and NLR in predicting the presence of resectable synchronous CRLM^24^-^26^, thereby strengthening prediction models. Neutrophils play a significant role in the progression and spread of CRLM, underscoring their clinical relevance. Neutrophils secrete interleukin-1 (IL-1) and tumor necrosis factor, stimulating the production of granulocyte-colony stimulating factors, resulting in elevated levels of vascular endothelial growth factor, antiapoptotic markers, and transcription factors^12^. Nevertheless, further research is necessary to fully elucidate the underlying mechanism. In a study by Pedrazzan^22^ *et al.*, it was revealed that patients with a high platelet count experience reduced long-term survival rates. The study further emphasized that a high platelet count independently acts as a prognostic factor for patients who underwent potentially curative resections, supporting our own findings. Despite these similarities, the exact mechanism remains uncertain, with several potential explanations requiring empirical validation^27^-^30^. Multiple studies have demonstrated that D-Dimer affects cellular communication systems, promoting increased cell proliferation and initiating angiogenesis^31^. Additionally, it has been observed to facilitate the progression and spread of malignancies by enhancing tumor cell attachment to endothelial cells^32^. Chen *et al.*^32^ have shown a correlation between elevated D-Dimer levels and decreased OS and DFS, consistent with our own research results. Furthermore, we incorporated D-Dimer levels into the clinical prediction model.

Our clinical predictive models offer several advantages. Firstly, patients with resectable synchronous CRLM were classified into high-risk and low-risk groups based on optimal cutoff values. Substantial differences in OS and DFS were noted across the risk categories. By using a risk score, physicians can tailor treatment strategies for resectable synchronous CRLM patients, aiding in the decision-making process between upfront surgical intervention and neoadjuvant therapy. Secondly, our nomograms incorporate a range of factors, including clinicopathological characteristics, genetic status, radiological findings, and preoperative blood parameters, providing a comprehensive evaluation. Moreover, the prognostic factors considered are readily available through routine preoperative examinations and blood tests, enabling healthcare professionals to personalize treatment plans for each patient.

Nevertheless, our study does have limitations. The retrospective design of the research poses a constraint that could lead to recall bias. Implementing a multicenter prospective cohort study would offer further evidence to substantiate our model. Moreover, to mitigate selection bias, we excluded patients with ambiguous data in their variables. For additional assurance, it may be necessary to validate the nomograms employed in this study through confirmation from cohorts at diverse medical centers.

## Conclusion

Our study was groundbreaking as it identified nine preoperatively accessible indicators from four critical domains: clinicopathological features, gene status, imaging, and hematology factors. These indicators were instrumental in developing a predictive model specifically tailored for direct surgery in resectable synchronous CRLM patients. The model has demonstrated exceptional accuracy in guiding treatment selection. In summary, we present a novel predictive model that estimates the survival rates of resectable synchronous CRLM patients who have undergone direct surgery using easily obtainable clinical indicators. This tool is anticipated to significantly aid clinicians in treatment decision-making processes between upfront surgery and neoadjuvant therapy.

## Supplementary Material

Supplementary figures and tables.

## Figures and Tables

**Table 1:** Characteristics of resectable synchronous CRLM patients

Characteristics, n (%)	Total cohort (n = 386)	Train cohort (n = 270)	Test cohort (n=116)
The largest metastatic tumor size (cm)			
≤2.250	166 (43.0)	113 (41.9)	53 (45.7)
>2.250	220 (57.0)	157 (58.1)	63 (54.3)
Number of metastatic tumors			
<3	281 (72.8)	204 (75.6)	77 (66.4)
≥3	105 (27.2)	66 (24.4)	39 (33.6)
cT stage			
cT1	6 (1.6)	6 (2.2)	0 (0.0)
cT2	17 (4.4)	14 (5.2)	3 (2.6)
cT3	213 (55.2)	147 (54.4)	66 (56.9)
cT4	150 (38.9)	103 (38.1)	47 (40.5)
cN stage			
cN0	111 (28.8)	81 (30.0)	30 (25.9)
cN1	146 (37.8)	95 (35.2)	51 (44.0)
cN2	129 (33.4)	94 (34.8)	35 (30.2)
KRAS gene			
Wild type	286 (74.1)	193 (71.5)	93 (80.2)
Mutant	100 (25.9)	77 (28.5)	23 (19.8)
NRAS gene			
Wild type	363 (94.0)	253 (93.7)	110 (94.8)
Mutant	23 (6.0)	17 (6.3)	6 (5.2)
BRAF gene			
Wild type	369 (95.6)	259 (95.9)	110 (94.8)
Mutant	17 (4.4)	11 (4.1)	6 (5.2)
Sex			
Male	235 (60.9)	166 (61.5)	69 (59.5)
Female	151 (39.1)	104 (38.5)	47 (40.5)
Age (years)			
≤70	221 (57.3)	153 (56.7)	68 (58.6)
>70	165 (42.7)	117 (43.3)	48 (41.4)
BMI			
Underweight	12 (3.1)	9 (3.3)	3 (2.6)
Normal weight	199 (51.6)	139 (51.5)	60 (51.7)
Overweight	131 (33.9)	93 (34.4)	38 (32.8)
Obesity	44 (11.4)	29 (10.7)	15 (12.9)
ASA			
I-II	350 (90.7)	239 (88.5)	111 (95.7)
III-IV	36 (9.3)	31 (11.5)	5 (4.3)
Differentiation grade			
Highly	331 (85.8)	229 (84.8)	102 (87.9)
Moderately and poorly	55 (14.2)	41 (15.2)	14 (12.1)
Pathological type			
Adenocarcinoma	381 (98.7)	267 (98.9)	114 (98.3)
Mucinous denocarcinoma	4 (1.0)	2 (0.7)	2 (1.7)
High-grade intraepithelial neoplasia	1 (0.3)	1 (0.4)	0 (0.0)
Original site			
Left semicolon	289 (74.9)	202 (74.8)	87 (75.0)
Right semicolon	97 (25.1)	68 (25.2)	29 (25.0)
CEA (ng/ml)			
≤ 16.110	245 (63.5)	172 (63.7)	73 (62.9)
> 16.110	141 (36.5)	98 (36.3)	43 (37.1)
Neutrophil count (*10^9^/L)			
≤ 3.435	154 (39.9)	110 (40.7)	44 (37.9)
> 3.435	232 (60.1)	160 (59.3)	72 (62.1)
Lymphocyte count (*10^9^/L)			
≤ 2.795	351 (90.9)	246 (91.1)	105 (90.5)
> 2.795	35 (9.1)	24 (8.9)	11 (9.5)
Monocyte count (*10^9^/L)			
≤ 0.545	290 (75.1)	204 (75.6)	86 (74.1)
> 0.545	96 (24.9)	66 (24.4)	30 (25.9)
Platelet count (*10^9^/L)			
≤ 282.510	302 (78.2)	213 (78.9)	89 (76.7)
> 282.510	84 (21.8)	57 (21.1)	27 (23.3)
NLR			
≤2.677	267 (69.2)	188 (69.6)	79 (68.1)
>2.677	119 (30.8)	82 (30.4)	37 (31.9)
PLR			
≤130.481	204 (52.8)	150 (55.6)	54 (46.6)
>130.481	182 (47.2)	120 (44.4)	62 (53.4)
CRP (mg/L)			
≤ 0.085	159 (41.2)	100 (37.0)	59 (50.9)
> 0.085	227 (58.8)	170 (63.0)	57 (49.1)
Albumin (g/L)			
≤ 44.950	269 (69.7)	183 (67.8)	86 (74.1)
> 44.950	117 (30.3)	87 (32.2)	30 (25.9)
RDW-CV			
≤ 15.350 %	291 (75.4)	205 (75.9)	86 (74.1)
> 15.350 %	95 (24.6)	65 (24.1)	30 (25.9)
D-Dimer (mg/L)			
≤ 0.545	257 (66.6)	180 (66.7)	77 (66.4)
> 0.545	129 (33.4)	90 (33.3)	39 (33.6)
GPS			
CRP≤10mg/L & Alb≥35g/L	374 (96.9)	262 (97.0)	112 (96.6)
CRP≤10mg/L & Alb<35g/L CRP>10mg/L & Alb≥35g/L	12 (3.1)	8 (3.0)	4 (3.4)
CRP>10mg/L & Alb<35g/L	0 (0.0)	0 (0.0)	0 (0.0)

**Table 2:** Cox regression analysis of OS in the training cohort

Factor	Univariate analyses		Multivariate analyses	
	*P* value	HR (95%CI)	*P* value	HR (95%CI)
The largest metastatic tumor size (cm)	0.457			
≤2.250		Reference		
>2.250		0.86 (0.58-1.28)		
Number of metastatic tumors	**<0.001**		**<0.001**	
<3		Reference		Reference
≥3		2.6 (1.69-4.01)		2.46 (1.54-3.92)
cT stage				
cT1		Reference		
cT2	0.520	0.45 (0.04-5.02)		
cT3	0.955	0.95 (0.13-6.86)		
cT4	0.790	0.79 (0.18-9.51)		
cN stage				
cN0		Reference		
cN1	0.057	1.72 (0.98-3.01)	0.590	1.18 (0.65-2.14)
cN2	**0.001**	3.05 (1.77-5.26)	**<0.001**	3.18 (1.82-5.57)
KRAS gene	**0.005**		**0.029**	
Wild type		Reference		Reference
Mutant		1.85 (1.21-2.83)		1.66 (1.05-2.71)
NRAS gene	0.391			
Wild type		Reference		
Mutant		1.48 (0.6-3.66)		
BRAF gene	**<0.001**		**<0.001**	
Wild type		Reference		Reference
Mutant		5.46 (2.36-12.66)		4.58 (1.88-11.17)
Sex	0.538			
Male		Reference		
Female		1.14 (0.76-1.7)		
Age (years)	**0.002**		**0.024**	
≤70		Reference		Reference
>70		1.89 (1.27-2.83)		1.62 (1.07-2.46)
BMI				
Underweight		Reference		
Normal weight	0.285	2.16 (0.53-8.85)		
Overweight	0.556	1.54 (0.37-6.48)		
Obesity	0.359	2.04 (0.45-9.32)		
ASA	0.143			
I-II		Reference		
III-IV		0.54 (0.24-1.23)		
Differentiation grade	0.648			
Highly		Reference		
Moderately and poorly		1.15 (0.63-2.12)		
Pathological type				
Adenocarcinoma		Reference		
Mucinous denocarcinoma	0.996	0 (0-Inf)		
High-grade intraepithelial neoplasia	0.998	0 (0-Inf)		
Original site	**0.039**		**0.046**	
Left semicolon		Reference		Reference
Right semicolon		1.59 (1.02-2.47)		1.61 (1.01-2.57)
CEA (ng/ml)	**0.016**		0.068	
≤ 16.110		Reference		Refernece
> 16.110		1.65 (1.1-2.49)		1.49 (0.97-2.28)
Neutrophil count (*10^9^ /L)	**0.089**		**0.010**	
≤ 3.435		Reference		Reference
> 3.435		1.46 (0.94-2.24)		1.80 (1.15-2.82)
Lymphocyte count (*10^9^ /L)	0.184			
≤ 2.795		Reference		
> 2.795		0.57 (0.25-1.31)		
Monocyte count (*10^9^ /L)	0.215			
≤ 0.545		Reference		
> 0.545		1.31 (0.85-2.01)		
Platelet count (*10^9^ /L)	**0.005**		**0.003**	
≤ 282.510		Reference		Reference
> 282.510		2.32 (1.29-4.16)		2.53 (1.37-4.68)
NLR	0.848			
≤2.677		Reference		
>2.677		1.04 (0.67-1.61)		
PLR	0.947			
≤130.481		Reference		
>130.481		0.99 (0.66-1.48)		
CRP (mg/L)	0.176			
≤ 0.085		Reference		
> 0.085		1.33 (0.88-2.01)		
Albumin (g/L)	0.339			
≤ 44.950		Reference		
> 44.950		0.61 (0.22-1.67)		
RDW-CV	0.316			
≤ 15.35 %		Reference		
> 15.35 %		1.26 (0.8-2)		
D-Dimer (mg/L)	**0.059**		**0.034**	
≤ 0.545		Reference		Reference
> 0.545		1.49 (0.98-2.25)		1.62 (1.04-2.51)
GPS				
CRP≤10mg/L & Alb≥35g/L		Reference		
CRP≤10mg/L & Alb<35g/L CRP>10mg/L & Alb≥35g/L	0.533	1.38 (0.5-3.75)		
CRP>10mg/L & Alb<35g/L	NULL	NULL		

**Table 3:** Cox regression analysis of DFS in the training cohort

Factor	Univariate analyses		Multivariate analyses	
	*P* value	HR (95%CI)	*P* value	HR (95%CI)
The largest metastatic tumor size (cm)	0.744			
≤2.250		Reference		
>2.250		0.94 (0.63-1.4)		
Number of metastatic tumors	**<0.001**		**<0.001**	
<3		Reference		Reference
≥3		2.47 (1.6-3.8)		2.48 (1.54-3.92)
cT stage				
cT1		Reference		
cT2	0.661	0.58 (0.05-6.45)		
cT3	0.838	1.23 (0.17-8.92)		
cT4	0.583	1.74 (0.24-12.65)		
cN stage				
cN0		Reference		
cN1	**0.020**	1.94 (1.11-3.4)	**0.193**	1.53 (0.82-2.63)
cN2	**<0.001**	3.14 (1.82-5.41)	**<0.001**	3.32 (1.89-5.92)
KRAS gene	**0.002**		**0.028**	
Wild type		Reference		Reference
Mutant		1.95 (1.27-2.98)		1.74 (1.06-2.67)
NRAS gene	0.643			
Wild type		Reference		
Mutant		1.24 (0.5-3.05)		
BRAF gene	**<0.001**		**0.032**	
Wild type		Reference		Reference
Mutant		5.85 (2.52-13.57)		2.81 (1.09-7.14)
Sex	0.752			
Male		Reference		
Female		1.07 (0.71-1.6)		
Age (years)	**0.004**		**0.022**	
≤70		Reference		Reference
>70		1.8 (1.2-2.69)		1.62 (1.07-2.51)
BMI				
Underweight		Reference		
Normal weight	0.348	1.96 (0.48-8.05)		
Overweight	0.725	1.29 (0.31-5.45)		
Obesity	0.570	1.55 (0.34-7.09)		
ASA	0.154			
I-II		Reference		
III-IV		0.55 (0.24-1.25)		
Differentiation grade	0.639			
Highly		Reference		
Moderately and poorly		1.16 (0.63-2.12)		
				
Original site	**0.037**		**0.021**	
Left semicolon		Reference		Reference
Right semicolon		1.6 (1.03-2.48)		1.83 (1.09-2.79)
CEA (ng/ml)	**0.006**		0.069	
≤ 16.110		Reference		Refernece
> 16.110		1.78 (1.18-2.69)		1.51 (0.97-2.27)
Neutrophil count (*10^9^ /L)	**0.041**		**0.044**	
≤ 3.435		Reference		Reference
> 3.435		1.43 (1.03-2.42)		1.61 (1.01-2.52)
Lymphocyte count (*10^9^ /L)	0.165			
≤ 2.795		Reference		
> 2.795		0.56 (0.24-1.27)		
Monocyte count	**0.088**		0.197	
≤ 0.545		Reference		Reference
> 0.545		1.45 (0.95-2.23)		1.41 (0.85-2.14)
Platelet count	**0.005**		**0.003**	
≤ 282.5		Reference		Reference
> 282.5		2.33 (1.29-4.18)		2.58 (1.38-4.70)
NLR	0.909			
≤2.677		Reference		
>2.677		0.97 (0.63-1.51)		
PLR	0.841			
≤130.481		Reference		
>130.481		0.96 (0.64-1.44)		
CRP (mg/L)	0.190			
≤ 0.085		Reference		
> 0.085		1.32 (0.87-1.99)		
Albumin (g/L)	0.218			
≤ 44.950		Reference		
> 44.95 0		0.53 (0.2-1.45)		
RDW-CV	0.425			
≤ 15.350 %		Reference		
> 15.350 %		1.2 (0.76-1.9)		
D-Dimer (mg/L)	**0.055**		**0.041**	
≤ 0.545		Reference		Reference
> 0.545		1.5 (0.99-2.27)		1.62 (1.02-2.48)
GPS				
CRP≤10mg/L & Alb≥35g/L		Reference		
CRP≤10mg/L & Alb<35g/L CRP>10mg/L & Alb≥35g/L	0.358	1.6 (0.59-4.37)		
CRP>10mg/L & Alb<35g/L	NULL	NULL		
