# Supplementary material for: Integrative Predictive Nomograms for Treatment Decision-Making in Resectable Synchronous Colorectal Liver Metastases
Source: J Cancer. 2025 Jan 27;16(5):1451–65. doi: 10.7150/jca.107194 (PMC11843234; doi:10.7150/jca.107194)
Supplement: Supplementary file 1 — Supplementary figures and tables. [file jcav16p1451s1.pdf]

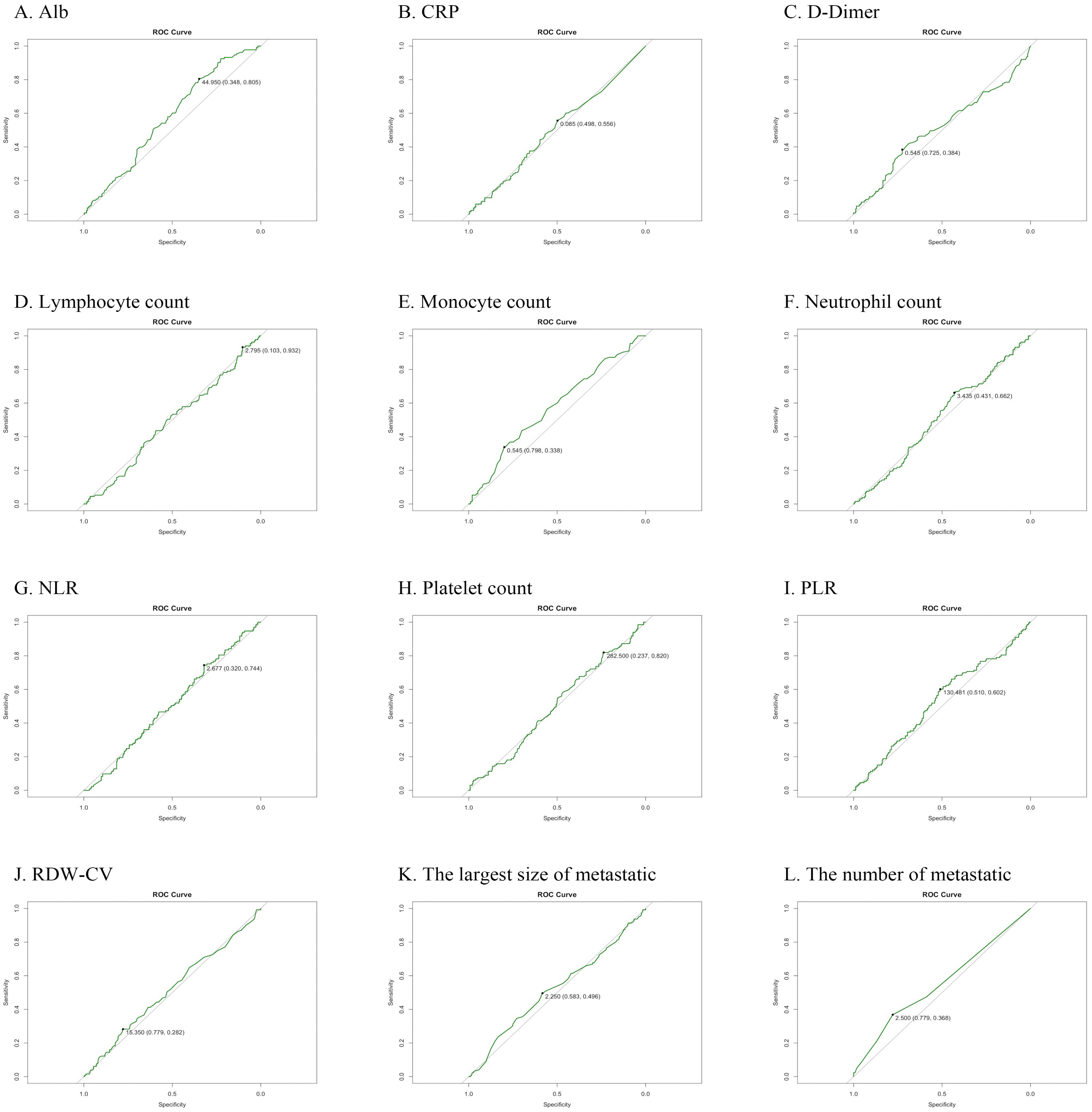

Supplementary Figure 1. Cut-off value obtained from the ROC curve

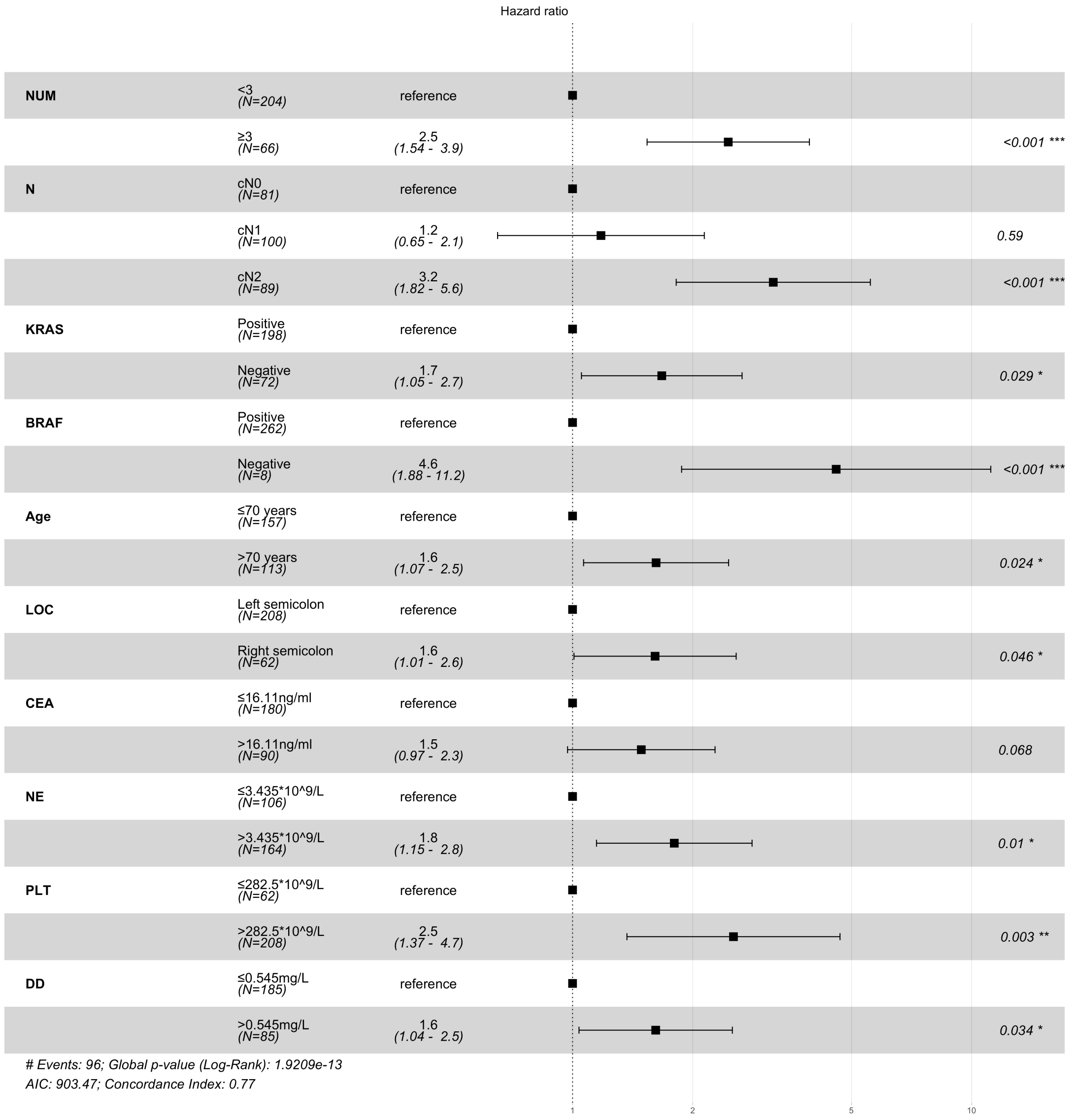

Supplementary Figure 2. forest plot for OS

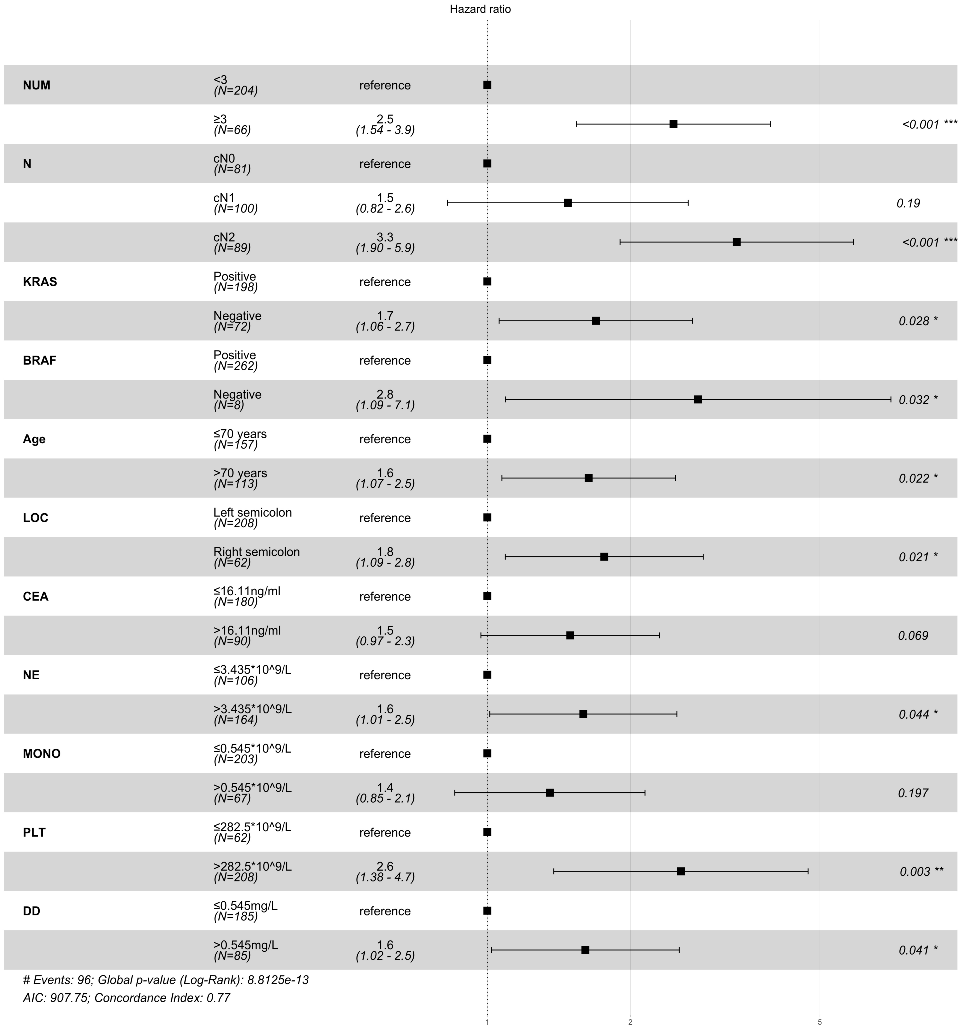

Supplementary Figure 3. forest plot for DFS
